# The Influence of Resistance Training on Joint Flexibility in Healthy Adults: A Systematic Review, Meta-analysis, and Meta-regression

**DOI:** 10.1519/JSC.0000000000005000

**Published:** 2024-12-31

**Authors:** Francesco Favro, Enrico Roma, Stefano Gobbo, Valentina Bullo, Andrea Di Blasio, Lucia Cugusi, Marco Bergamin

**Affiliations:** 1 Department of Medicine, University of Padova, Via Giustiniani, Padova, Italy;; 2 Univ Lyon, UJM Saint-Etienne, Laboratoire Interuniversitaire de Biologie de la Motricité (LIBM), Saint-Etienne, France;; 3 Department of Statistical Sciences “Paolo Fortunati”, University of Bologna, Bologna, Italy;; 4 Department of Human Movement Science, G. D'Annunzio University of Chieti-Pescara, Via dei Vestini, Chieti, Italy; and; 5 Department of Biomedical Sciences, University of Sassari, Sassari, Italy

**Keywords:** strength training, range of motion, stretching, PNF

## Abstract

Favro, F, Roma, E, Gobbo, S, Bullo, V, Di Blasio, A, Cugusi, L, and Bergamin, M. The influence of resistance training on joint flexibility in healthy adults: A systematic review, meta-analysis, and meta-regression. *J Strength Cond Res* 39(3): 386–397, 2025—Joint flexibility is a key component of physical fitness. Despite the large body of evidence regarding the effectiveness of muscle stretching exercises, little is known about the effect of resistance training on flexibility. A systematic search was conducted on 9 academic search instruments; inclusion criteria were as follows: healthy adult participants (age ≥18 years); resistance training intervention (duration ≥4 weeks); at least one flexibility outcome. Risk of bias was assessed using the RoB-2 and ROBINS-I tools. A 3-level meta-analysis was conducted, with multiple outcomes nested within each study. A moderator analysis was conducted by fitting a meta-regression model. Significance level was set at *p* < 0.05. We included 36 studies (1,469 participants). None of the included papers resulted at a low risk of bias. The pooled effect size for resistance training on flexibility was *g* = 0.6325, with 95% CI: 0.4762 to 0.7888 (*p* < 0.0001). There was a substantial amount of heterogeneity between studies. Exercise intensity was a significant moderator (*p* < 0.0225, high vs low), based on 129 and unique effect sizes, and sex (*p* = 0.0429). Activity level and age were nonsignificant moderators. Resistance training could be implemented as a strategy to improve joint flexibility, with high-intensity protocols resulting in a magnified effect. However, the high overall risk of bias and substantial heterogeneity limit our ability to draw definitive conclusions.

## Abstract 2

Supplemental Digital Content is Available in the Text.

## Introduction

Flexibility can be defined as the ability to move a joint through its full range of motion (21), as allowed by the intrinsic mechanical properties of body tissues (60) and stretch tolerance (53). Joint flexibility is considered a key component of physical fitness (86), and the American College of Sports Medicine physical activity guidelines refer to flexibility exercises as an appropriate component of a physical activity program for older adults, although they note that flexibility training by itself does not demonstrably reduce the risk of injury and that these activities do not contribute toward meeting the aerobic or muscle-strengthening recommendations (64).

In the current literature, there are 2 main theories that explain how a chronic muscle stretching program improves flexibility. The mechanical theory (52) assumes changes in the mechanical properties of tendons and muscles, focusing on factors such as the stiffness of the muscle-tendon unit to explain the improvement in flexibility. By contrast, the sensory theory (27,94) attributes the increase in flexibility to a higher maximum tolerated passive torque, also known as stretch tolerance (53). Freitas et al. (22) concluded in their meta-analysis that short- to medium-term stretching programs (up to 8 weeks of duration) result only in sensory level adaptations, measured as an increase in the maximal tolerated passive torque (SMD = 0.54, 95% CI = 0.15–0.92), with trivial effects on muscle (SMD = −0.19, 95% CI = −0.77 to 0.39) and tendon (SMD = −0.06, CI = −0.32 to 0.20) stiffness, although the selected studies were highly heterogeneous, and adaptations to the muscle-tendon unit (MTU) might require longer training durations. It is important to note, however, that in the same meta-analysis, the 2 included PNF studies both reported a decrease in tendon stiffness (44,54). Two more recent meta-analyses, both published in 2023 by Takeuchi et al. (84,85) concluded instead that static stretching is effective in decreasing the stiffness of the muscle-tendon unit but not that of the muscle alone.

Proprioceptive neuromuscular facilitation (PNF) is a particular stretching technique that is composed of cycles of stretching, contraction, and relaxation, to reach a greater ROM. When performed at sufficient intensity, with the literature suggesting intensities of 65–70% of MVIC (50,75), this stretching technique could be seen as showing similarities with isometric resistance training performed in a lengthened position. Other modalities of training can also induce increases in flexibility: eccentric resistance training can increase the fascicle length of the muscles and the ROM of the joints involved while concurrently increasing strength (1,58,66). However, the effect of concentric and isotonic resistance training on joint flexibility is still debated, with some of the literature indicating an improvement in ROM (2,10,76), and a few articles that report no significant effect (53).

A possible explanation for an improvement in flexibility after a resistance training protocol could be the similarity with the PNF technique (especially when performing relatively intense isometric contractions, as noted above) when an exercise is performed through the joints' full ROM because the muscles involved are contracting while fully lengthened (e.g., during a dumbbell bench press exercise, the pectoralis major reaches complete lengthening at the bottom of the movement, in a state of maximal contraction), which might elicit a similar response. Furthermore, a possible mechanism for improved flexibility in aged, sedentary persons after a resistance training intervention could be an effect of exercise in countering the stiffening of tissue occurring because of the aging process and disuse (12); animal studies report an increase in cross-linking within collagen filaments with age (26) and a decrease in the elasticity of tendons, with a concurrent increase in tissue viscosity, which can be counteracted in older subjects with exercise training (47).

A review by Nuzzo (54), debating the place of flexibility as a major component of physical fitness, showed extensive data supporting the hypothesis that resistance training, as an isolated intervention or combined with aerobic or stretching exercises, could have a positive effect on joint flexibility. Nuzzo, however, only collected and presented the data without pooling it with meta-analytical techniques. After this review, a systematic review and meta-analysis (3), screening 194 records, compared the effect of resistance training vs stretching interventions and found a similar effect on flexibility for both training modalities (slightly favoring stretching, ES = −0.22, 95% CI = −0.55 to 0.12; *p* = 0.206), although the authors did not exclude participants based on their health status or age, including, for example, individuals with fibromyalgia, that commonly results in joints stiffness and reduced ROM (57), which could therefore introduce a confounding factor on the analysis. In another recent review (5), Alizadeh et al. concluded that resistance training has a significant effect on ROM (ES = 0.73; *p* < 0.001) when compared with a control group and found, similarly to Afonso et al., that resistance training has a comparable effect to stretching interventions (ES = 0.08; *p* = 0.79). In their work, the authors performed subgroup analyses, but did not include training variables such as intensity, volume, or frequency as moderators; such variables are an important source of heterogeneity between RT studies, are central to determine a prescription of RT, and play a large role in determining its effects on strength and hypertrophy (see, for example, Refs. 67,68), and as such, we can presume that they would play a role in moderating the effect on joint flexibility as well. For these reasons, we believe that further investigations are needed to add clarity on the subject of resistance training and joint flexibility.

Therefore, the aim of this systematic review with meta-analysis and meta-regression was to evaluate the effect of resistance training (RT) on joint flexibility in healthy adults and the moderating effects of study-, sample- and intervention-level variables. Our hypothesis is that resistance training alone can lead to increases in flexibility compared with a control condition and that isolated RT interventions will show a greater effect on older or sedentary individuals rather than younger or active persons.

## Methods

### Experimental Approach to the Problem

A systematic review and 3-level meta-analysis was conducted, using a meta-regression approach to assess the influence of moderating variables on the outcome of interest. The review was approved by the University of Padova Institutional Review Board.

### Search Strategy

A systematic search was conducted (PROSPERO REGISTRATION NUMBER: CRD42021242692) on 9 academic search instruments (PubMed, Scopus, SPORTDiscus, CINAHL, PEDro, Web of Science, VHL, EMBASE, and SpringerLink). The search instruments were chosen after evaluating their coverage, main subject, and search options among the 28 analyzed by Gusenbauer et al. (28). The search string was built from relevant Mesh Terms, additional terms found in prominent works in the fields of resistance training and flexibility research (51,60,73,74,93), and from our knowledge, after a brainstorming session (13). The full search string for PubMed, available in Supplemental Digital Content 1 (see Content A, http://links.lww.com/JSCR/A556), was then adapted for, and run on: Scopus, SPORTDiscus, CINAHL, PEDro, Web of Science (WoS), Virtual Health Library (VHL), Bielefeld Academic Search Engine (BASE), and SpringerLink. The results were then imported into Mendeley Desktop to detect and remove duplicates (48). In addition, backward and forward citation searching on PubMed and Scopus from included articles was performed to search for additional records. An additional round of searches and data extraction was performed in February 2022 using the same methods, and other eligible articles were retrieved in April 2023 from the bibliography of Alizadeh et al. (5).

### Eligibility Criteria

Following the PICO model (72), we included studies with the following characteristics: (a) Age ≥18 years; (b) Healthy participants; (c) Resistance training intervention for at least 4 weeks; (d) Any measure of ROM or flexibility as outcome. And excluded studies based on: (a) Age <18 years; (b) Participants with any adverse health conditions; (c) No isolated resistance training intervention or intervention duration <4 weeks; (d) No measure of ROM or flexibility.

### Screening Process

The first abstract screening process was carried out from November 2020 to January 2021 on Rayyan.qcri.org (now rayyan.ai), a free web and mobile application (61), which was also used to double check the deduplication performed in Mendeley. Papers that passed the first screening were read in full to assess their eligibility.

### Data Extraction

Relevant data were extracted from the included articles in an adapted version of the Cochrane standardized extraction form (https://dplp.cochrane.org/data-extraction-forms) and included.

- Study identifiers: title, first author, corresponding author, year, country, and study ID.
- Eligibility, based on the PICO model, with eventual reasons for exclusion.
- Study characteristics: aim, design, randomization, unit of allocation, start or end date, duration, ethical approval, and informed consent.
- Participants: description, inclusion or exclusion criteria, method of recruitment, numerosity, age, sex, training status, baseline imbalances, and other sociodemographic data.
- Type of intervention(s): volume, intensity, rest, progression, frequency, delivery, providers, and duration (weeks) of the intervention.
- Comparison group(s): intervention(s) or control group data.
- Withdrawals and exclusions, with reasons, and adverse events.
- Main outcome and secondary outcome measurements (method and number of measures, blinding of the researchers, intention-to-treat analysis, time points reported, unit of measure).
- Mean and SD for each outcome (or other measures, such as median, standard error, interquartile range, and confidence intervals) and other results reported (*p* values, effect sizes, mean changes, preintervention to postintervention correlation, etc).
- Study funding, conflicts of interest, references to other relevant studies, and main conclusions of the authors.

### Risk of Bias Assessment

In addition, risk of bias was assessed using the RoB2 and ROBINS-I tools for randomized and nonrandomized interventions, respectively (81,82). The tool is used to analyze the risk of bias separately for each outcome included in each paper. To streamline the process, in papers that measured the same, or very similar outcomes for different body regions (e.g., 5RM of chest press, leg press, and lat pull-down, or goniometric assessment of knee flexion, hip flexion, and shoulder flexion), we aggregated the risk of bias analysis. A similar process was carried out when using the ROBINS-I tool.

This tool provides the overall judgement based on the worst score among the various domains analyzed. Furthermore, questions 2.1 and 2.2, regarding blinding of participants and researchers were hardly relevant, as blinding of participants is impossible in physical exercise interventions. Finally, we have to note that the RoB2 guidelines accept a “modified intention-to-treat analysis” to award a positive score in the appropriateness of the analysis used (question 2.5), which meant that papers that excluded participants who did not complete the intervention from both the preintervention and the postintervention data were scored positively, and the studies where no dropouts were reported.

### Statistical Analyses

Similar RT groups within the same study were combined for each time point using the equations in the Cochrane handbook, table 6.5 a (31): the sample size of the combined group was calculated as:

(1) $$n_{i,h,t}=n_{1,i,t}+n_{2,i,t},$$

the estimated mean of the outcome as:

(2) $$\hat{\mu_{i,j,h,t}}=\frac{n_{1,i,h,t}\cdot \hat{\mu_{1,i,j,h,t}}+n_{2,i,h,t}\cdot \hat{\mu_{2,i,j,h,t}}}{n_{1,i,h,t}+n_{2,i,h,t}}$$

and the estimated standard deviation as:

(3) $$\hat{\sigma_{i,j,h,t}}=\sqrt{\frac{\left(n_{1,i,h,t}-1\right)\hat{\sigma_{1,i,j,h,t}^{2}}+\left(n_{2,i,h,t}-1\right)\hat{\sigma_{2,i,j,h,t}^{2}}+\frac{n_{1,i,h,t}\cdot n_{2,i,h,t}}{n_{1,i,h,t}+n_{2,i,h,t}}\left(\hat{\mu_{1,i,j,h,t}^{2}}+\mu_{2,i,j,h,t}^{2}-2\hat{\;\mu_{1,i,j,h,t}\cdot }\hat{\mu_{2,i,j,h,t}}\right)}{n_{1,i,h,t}+n_{2,i,h,t}-1}}$$

where *n*, $\hat{\mu }$, and $\hat{\sigma }$ are, respectively, the sample size, the estimated mean and *SD* of the combined group, for the study *i*, the outcome *j*, the group *h* (resistance training or control groups), and the time *t* (preintervention or postintervention).

All preintervention and postintervention outcome data were converted or estimated into mean and *SD*; specifically, to estimate the mean and *SD*. of the “flexitest” outcomes in “2005_Nòbrega,” the methods outlined by Wan et al. (92) were used. Where *SD* were unavailable, the authors were contacted, and, if the correspondence was unsuccessful, imputation of *SD* was obtained from data on the same tests in similar populations.

Effect sizes (ES) for each outcome and their variances were computed using the standardized mean change using raw score standardization (Equations 4–6) as described by Becker and Morris (11,55) because all included articles used pretest and posttest case-control designs. The effect size was calculated as:

(4) $$g_{ij}=c_{i,T}\cdot \frac{\hat{\mu_{ijT,post}}-\hat{\mu_{ijT,pre}}}{\hat{\sigma_{ij,Tpre}}}-c_{i,C}\frac{\hat{\mu_{ijC,post}}-\hat{\mu_{ijC,pre}}}{\hat{\sigma_{ij,Cpre}}}$$

where *g* is Hedge's *g*, $\hat{\mu }$ and $\hat{\sigma }$ are the mean and *SD* of the treatment group, *T*, and control group, C, measured at preintervention, pre, and postintervention, post, whereas *c* is a bias correction factor, defined for each study and group as:

(5) $$C_{i,h}=1-\frac{3}{4\left(n_{i,h}-1\right)}$$

The variance of the effect size *g* was calculated as:

(6) $$\sigma_{gi,j}^{2}=c_{i,T}^{2}\left(\frac{2\left(1-{\hat{\rho }}_{i,j,T}\right)}{n_{i,T}}\right)\left(\frac{n_{i,T-1}}{n_{i,T-3}}\right)\left(1+\frac{n_{i,T}g_{i,j,T}^{2}}{2\left(1-{\hat{\rho }}_{i,j,T}\right)}\right)-g_{i,j,T}^{2}+c_{i,C}^{2}\left(\frac{2\left(1-{\hat{\rho }}_{i,j,C}\right)}{n_{i,C}}\right)\left(\frac{n_{i,C-1}}{n_{i,C-3}}\right)\left(1+\frac{n_{i,C}g_{i,j,C}^{2}}{2\left(1-{\hat{\rho }}_{i,j,C}\right)}\right)-g_{i,j,C}^{2}$$

where $\sigma^{2}$ is the variance of the effect size *g*, $\hat{\rho }$ is the estimated preintervention to postintervention correlation coefficient, and *c* is the correction factor defined in Equation 5.

Hedge's *g* is an unbiased estimator of the standardized mean difference. Hence, it can be interpreted using the same rule as the Cohen's *d*: a value of 0.2 is classically interpreted as small, 0.5 as medium, and 0.8 as a large effect (15). Caution is needed, however, because these values are dependent on the particular discipline and context (14). For example, Steele et al. reported in their meta-analysis of resistance training studies an average SMD of 0.87 for strength outcomes and of 0.34 for hypertrophy outcomes (80), whereas, to the best of our knowledge, no such data are available for flexibility outcomes.

To estimate ρ, the preintervention to postintervention correlation coefficient for each group was estimated directly for the studies in which sufficient data were reported, using the following formula (Equation 7), as reported in the Cochrane handbook (31):

(7) $${\hat{\rho }}_{i,j}=\frac{\hat{\sigma_{i,j,pre}^{2}}+\hat{\sigma_{i,j,post}^{2}}+\hat{\sigma_{i,j,change}^{2}}}{2\times \hat{\sigma_{i,j,pre}}\cdot \hat{\sigma_{i,j,post}}}$$

where the index change indicates the *SD* of preintervention to postintervention change scores.

When ρ could not be estimated, an imputation was made of *ρ* = 0.7, which was then tested by performing an iterative sensitivity analysis, computing the main model with different ρ values (from 0.1 to 0.9, with 0.1 step increases).

A 3-level meta-analysis model, with ES nested at the study level was fitted using the restricted maximum likelihood (REML) method. To test the variance-covariance structure of the model, the null hypothesis of model reduction (a two-level model without ES clustered within the studies) was tested using a likelihood ratio test, re-estimating both models with the maximum likelihood method.

The presence of heterogeneity was tested using the Cochran's *Q* statistics; however, this heuristic was not used to simplify the model. I^2^ statistic was reported to quantify the impact of heterogeneity and to allow between study comparisons. A rule of thumb to guide the interpretation of the I^2^ statistic, as given by the Cochrane handbook (6), is to consider values lower than 40% as not important, between 30 and 60% as moderate, between 50 and 90% as substantial, and between 75 and 100% as considerable heterogeneity (3). Multilevel decomposition of the *I*^2^ statistic was used to discriminate among between-cluster and within-cluster heterogeneity.

Prediction intervals (PIs) were calculated to represent the impact of heterogeneity on the Hedge's *g* scale. PIs are less sensitive to sample size and give a better understanding of the true dispersion of the effects for a random effects meta-analysis (35).

Moderator analysis was carried out by testing one variable at a time in a 3-level meta-regression model. Meta-regression (reported in full in see Content D, Supplemental Digital Content*,* http://links.lww.com/JSCR/A559) was conducted with the following study-level variables, tested separately: year, randomization, baseline differences (between RT and control groups) and unit of analysis, and the following descriptive variables relating to the participants and the intervention(s): treatment duration, sex, activity level, mean age, number of exercises, total volume, and volume per exercise, Accordance between the muscles trained and joints tested in the flexibility outcomes (Mismatch), progression, timing (number of sessions per week), delivery (supervised or not), intensity (grouped as low, moderate, or high), rest time between sets, and body parts tested. Year, treatment duration, mean age, number of exercises, total volume, volume per exercise, timing, and rest time were coded as continuous variables; randomization, baseline differences, unit of analysis, sex, activity level, mismatch, progression, delivery, intensity (grouped), and body parts tested were coded as categorical variables.

The presence of influential observations on model coefficients and variance component was tested using DFBETAs, and calculating model residuals and Cook's distances, according to Viechtbauer and Cheung (91). Because most of the studies also included strength outcomes (1RM, peak torque, etc.), a 3-level meta-analysis was conducted for strength as well, using the same methodology (excluding the meta-regression) followed for the main analysis, to verify the effectiveness of the protocols proposed. The statistical software R, version 4.3.2 (67), and the packages metafor, version 4.4.0 (90), and tidyverse, version 2.0.0 (96) were used to conduct all statistical analyses and draw all included graphs.

## Results

**Figure 1.**
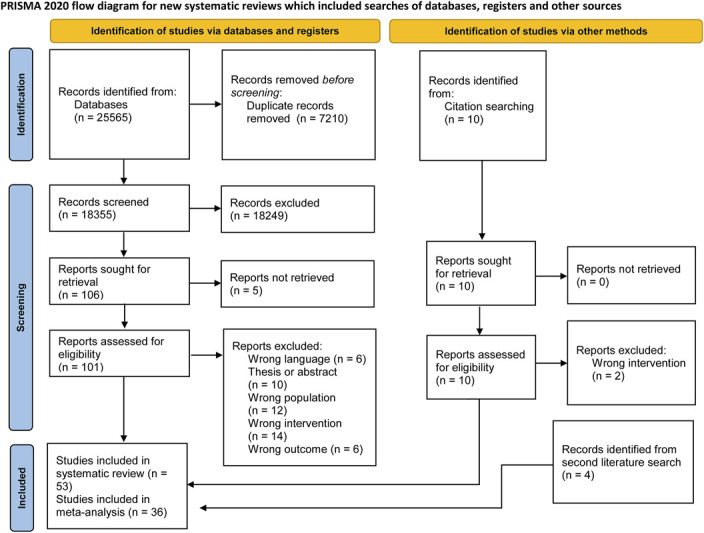
PRISMA flow diagram.

The systematic search resulted in 25,565 items, with 18,355 remaining after deduplication. The abstract screening process resulted in 106 articles included for full-text screening, with 4 additional articles retrieved by backward and forward citation searching. Fifty-five (2 of which from citation searching) articles met inclusion criteria, of which 26 were included in the statistical analysis and are summarized in Table 1 of Supplemental Digital Content (see Content B, http://links.lww.com/JSCR/A557). The full search strategy is reported in Figure 1 using the flow diagram structure from the PRISMA Statement (62). The additional search conducted in 2022 resulted in the inclusion of 4 articles, whereas 6 further articles were included from Alizadeh et al. (5), for a total of 36 articles and 169 unique effect sizes included in the meta-analysis.

Training groups were aggregated in the studies by Balogun et al. Fatouros et al., Jùnior et al. Kalapothrakos et al., and Leite et al. (9,20,37,38,49). Control groups were not aggregated in any of the included studies because the only study in which this would have been relevant, Baker et al. (8), featured walking as an additional group, which we deemed different enough from the “no activity” control group and was therefore excluded from all analyses.

Mean sample size (dropouts excluded) was 40.8 ± 20.4, with a total sample across all RT and control groups of 1,469 participants, with a mean age of 46.2 ± 23.1 years from 35 of the studies (data unavailable from Junior et al. (37)). Twenty-two studies included sedentary participants, 7 had active participants, and 7 did not provide information on their participants' activity levels. Mean intervention duration was 11.08 ± 6.16 weeks (range 4–24 weeks), with a mean frequency of 2.93 ± 0.90 sessions per week and a mean volume in each session of 20.27 ± 13.93 sets.

Exercise intensity was reported as 1RM% in only 12 studies, with 5 more reporting intensity as *n*RM (5RM, 8RM, etc) and 2 reporting it as a measure of perceived effort. Regarding flexibility outcomes, the sit-and-reach test, or its modified variation, was the most assessed (21 of 36 studies), followed by goniometric assessments of ROM (12 of 36 studies included at least one).

**Figure 2.**
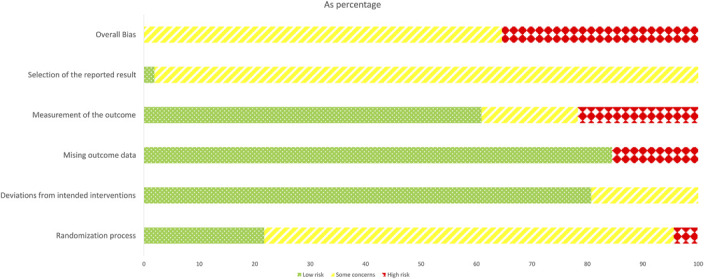
Risk of bias summary for randomized studies, reported as percentage of the total on the x-axis.

The risk of bias analysis for randomized trials (RoB2) revealed that none of the included papers were at a low risk of bias: 64.6% of the analyzed outcomes were judged as “some concerns,” whereas 35.4% were judged as “high” risk of bias (Figure 2, generated using the RoB2 excel tool, and Supplemental Digital Content C). The analysis for nonrandomized trials (ROBINS-I), instead, resulted in all 9 papers being judged as at least a “serious” risk of bias, with 3 (18,56,63) being judged at “critical” risk of bias (see table 2c, Supplemental Digital Content, http://links.lww.com/JSCR/A558).

The point estimate of the ES from the 36 studies included was *g* = 0.6325 ± 0.0797, with 95% confidence intervals: 0.4762–0.7888, and a *p* value <0.0001. The likelihood ratio test supports the rejection of the null hypothesis of model reduction and favors the 3-level model (*χ*^2^ = 29.56, *p* < 0.0001). According to the results of the Lilliefors normality test, model residuals seem to be not normally distributed (*D* = 0.1960, *p* < 0.0001), although no high Cook's distances or DFBETAs were detected. The results of the sensitivity analysis performed with different ρ imputations confirms the robustness of the chosen model. Multilevel variance decomposition reveals a total amount of heterogeneity *I*^2^_total_ = 69.3%, with a between-study (I^2^_level 3_) heterogeneity of 38.5%. 95% PI for the model, taking heterogeneity into account, are (−0.3463 to 1.6113).

Regarding the meta-regression model, sex was a weakly significant moderator (*p* = 0.496), with no differences between studies that included exclusively male or female subjects, and a lower estimate for studies that included both (0.3920 ± 0.1220, 95% CI: 0.1529 to 0.6311, 95% PI: −0.5572 to 1.3412).

**Figure 3.**
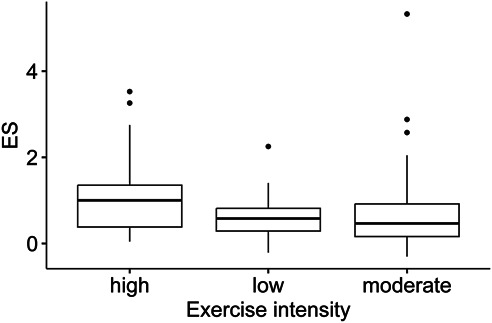
Exercise intensity grouped as “low”, “moderate”, and “high”. ES = Hedge’s g.

Among resistance training variables, exercise intensity, when grouped (Figure 3), was a significant moderator of the pooled ES, based on 129 unique ES (*p* value < 0.0225, high vs low): low intensity shows an ES = 0.2843 (95% CI: −0.0780 to 0.6466; 95% PI: −0.6936 to 1.2622), and high intensity an ES = 0.7495 (95% CI: 0.4960 to 1.0030; 95% PI: −0.1935 to 1.6925).

Rest time between sets (138 unique ES), was another significant moderator (intercept = 0.2788 ± 0.1315, rest = 0.0033 ± 0.0010, *p* = 0.0007). 95% PI were calculated for 3 rest times: 60 seconds: −0.4071 to 1.3554; 120 seconds: −0.2093 to 1.5484; 240 seconds: 0.1435 to 1.9773. A nonsignificant effect was found for activity level (*p* = 0.2543), with an estimate 0.7009 ± 0.1051 for sedentary participants and 0.4530 ± 0.1903 for physically active individuals, and for age (intercept = 0.5622 ± 0.1810, age = 0.0011 ± 0.0035, *p* = 0.7570).

There were 4 outcomes (from 2 studies: Fatouros et al. [20] and Lima Monteiro et al. [51]) with ES >3 (with the goniometric measure of trunk extension ROM from Monteiro_2008 resulting in an exceptionally high ES of 15.4). Removing the outcomes with an ES >3, the point estimate of the ES is lowered slightly, to 0.6178 ± 0.0787, with 95% confidence intervals: 0.4636 to 0.7721, and a *p* value <0.0001.

**Figure 4.**
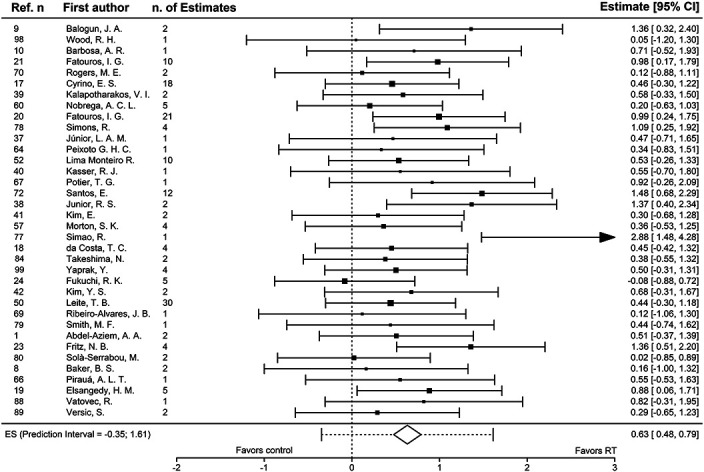
Forest plot, 95% CI: 95% confidence intervals, ES: standardized mean change with raw score standardization, n. of Estimates: the number of unique effect sizes nested within each study. Arrows in confidence intervals denote that they continue beyond the limits of the forest plot. Dotted lines indicate the prediction intervals.

This reduced model still shows a high amount of heterogeneity (I^2^_total_ = 67.71%), with an increased between-study heterogeneity I^2^_level 3_ = 42.36%. ES and variance were then aggregated at the study level to draw legible plots: Figure 4 shows the forest plot of the aggregated data.

**Figure 5.**
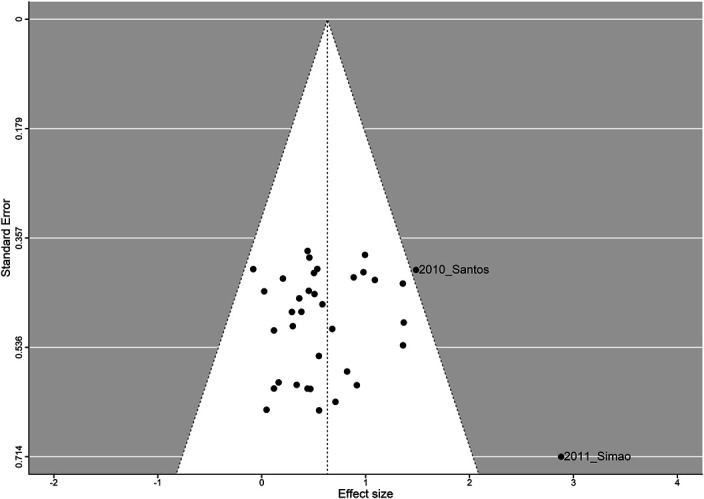
Funnel plot, centered on the pooled estimate. Every point represents the combined ES of all outcomes nested within each study.

**Figure 6.**
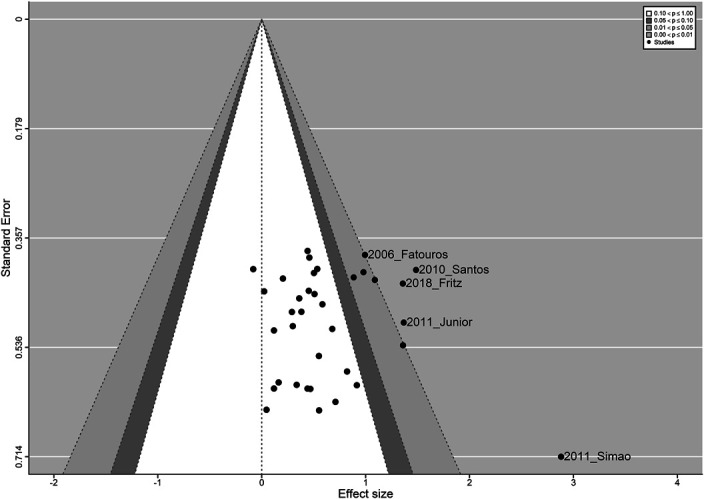
Contour-enhanced funnel plot, centered at 0. Every point represents the combined ES of all outcomes nested within each study; shaded areas represent different levels of significance.

**Figure 7.**
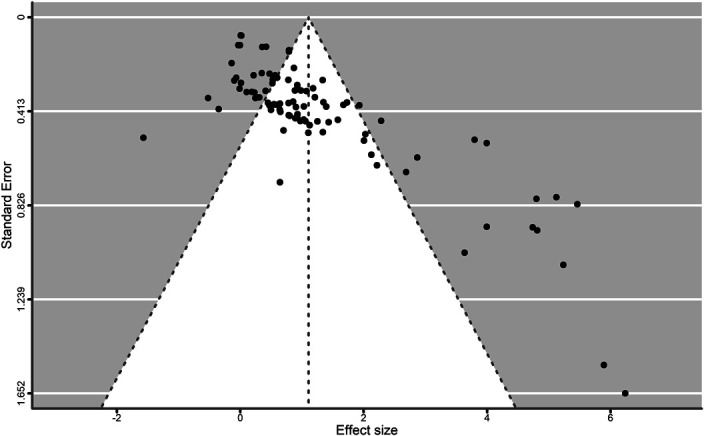
Strength outcomes funnel plot. Every point represents the combined ES of all outcomes nested within each study.

The point estimate for strength outcomes (94 outcomes from 26 studies) was 1.1056 ± 0.1957 (*g* ± *SE*), with 95% CI: 0.7220, 1.4892; *p* < 0.0001. The log-likelihood test and information criteria support the use of a 3-level model (*χ*^2^ = 28.99, *p* < 0.0001). Figures 5, 6 and 7 show the standard and contour-enhanced funnel plots for the aggregated flexibility data, and the funnel plot for strength data.

Upon visual inspection, Figures 5 and 6 reveal a slightly higher number of data points to the left of the overall ES, with 3 outlier studies, indicating that publication bias does not seem to be present in the included studies. This could be explained by the fact that flexibility outcomes were rarely the main focus of the included studies, and therefore, the lack of significant findings may have not been a factor in the publication process. Conversely, Figure 7 shows marked asymmetry in the strength data, with a distribution compatible with the presence of a small-study effect.

## Discussion

The initial question that guided this project was whether resistance training influenced joint flexibility. The positive overall $\hat{g}$ = 0.6325 (95% CI: 0.4762–0.7888) estimated from 169 outcomes nested within the 36 studies included supports the hypothesis of an effect of resistance training on joint flexibility in a healthy, adult population. The 95% confidence interval estimate can be considered wide, because of high heterogeneity between studies, and shows a medium to large effect, although the resulting 95% PI (−0.3463 to 1.6113) cross the reference line at 0, indicating that the large heterogeneity between studies (possibly because of different types of training protocols, flexibility tests, populations, and study durations) limits our ability to infer that future studies would find a positive effect of resistance training on joint flexibility.

Contrary to our initial hypotheses, neither activity level nor age seem to be significant predictors of our effect of interest, although in both cases there is a trend toward a greater effect in the direction we expected it to be (sedentary and older participants, respectively); these variables were reported in most of the included studies (activity level was reported in 29 of 36, age in 35 of 36), although the activity level classes were somewhat imbalanced, with 22 studies being conducted on sedentary participants, and only 7 on active individuals, meaning that there are, to a degree, limited data on the effects of RT on flexibility specifically for physically active individuals, with only Junior et al. (37) explicitly including participants with strength training experience. Future studies conducted on active persons may be warranted to confirm these effects.

An *I*^2^ value of 69.3% may represent substantial heterogeneity between studies (32), and the multilevel decomposition of *I*^2^ revealed that only 38.54% of the heterogeneity is attributable to variance between the studies. One possibility is that the large number of different flexibility tests introduced heterogeneity in the model; for example, flexibility of the hamstrings was measured with the sit and reach, modified sit and reach, chair sit and reach, 90–90 test, passive straight leg raise test, and dynamometric goniometry tests. We attempted to code this discrepancy as goniometric tests and tests with results measured in centimeter, but no significant difference was detected. To address this issue, a consensus statement could be written to elaborate a core outcome set (16) for the most commonly adopted flexibility tests or a limited number of preferred tests for each joint (or series of joints).

The effect on flexibility seems to be particularly significant when performing high-intensity resistance training compared with low-intensity RT, based on 129 unique ES, nested within 23 studies. A meta-analytic study (74) have shown that high-intensity resistance training has a greater effect on muscle strength (based on 84 ESs from 14 studies, ES: 0.58, 95% CI: 0.28 to 0.89, favoring high loads), probably because it elicits greater motor unit recruitment and subjects the muscle fibers to greater mechanical tension (46). The physiological reasons underlying the greater effect on flexibility could be similar, or because of different activation of muscle spindles, although, to our knowledge, no study have investigated the effect of different exercise intensities on these sensory organs. In addition, high-intensity training could simply have a greater effect because it imposes higher internal loads compared with low-intensity training. For example, RT promotes better intermuscular coordination, that is, an improvement in coactivation patterns between agonists and antagonists muscles (29,33), which is part of the reason for the initial large increase in strength observed in RT novices, and that could aid in the expression of greater active ranges of motion because the antagonist muscles do not contract as much during a movement (which would require a higher strength expression from the agonist muscles). When training at high intensity, generally, compound exercises are chosen, which may promote greater neuromuscular coordination and have a positive effect on joint flexibility through a better agonist or antagonist coordination and relaxation patterns. Furthermore, resistance training also promotes an acute increase in pain tolerance, which is linked closely to stretch tolerance (7), and as such, after a RT intervention, the muscle-tendon unit could be moved through a greater ROM, even if the stiffness of the muscle-tendon unit remains unchanged before having to voluntarily ending the movement (because of reaching the limit of tolerance to the stretching sensation). Another significant moderator was rest time between sets, although higher rest times are usually used with higher intensities, which would mean that the variable is a proxy for exercise intensity.

Sex was shown to be a significant moderator when comparing studies with only male or only female participants vs studies recruiting both sexes. We are currently unable to form a solid hypothesis on the reasons that could have driven this difference because there was no relevant difference in ES between male- and female-only studies (*g* = 0.76 for males and *g* = 0.80 for females). Our results contrast the subgroup analyses performed by Alizadeh et al. (5), who found no statistically significant differences between male, female, and mixed groups. The studies involving mixed groups seem to be of lower overall quality, which could have affected the results in this subgroup. Furthermore, it has been reported before that women show greater baseline flexibility and a greater improvement in joint flexibility in response to stretching interventions (42,43), which could have introduced a confounding factor in the studies that recruited mixed groups. As a last hypothesis, we might posit that in the mixed groups, female participants might have experienced a greater degree of social physique anxiety (the “anxiety that people experience in response to others' evaluations of their physiques”), which is negatively correlated to physical activity participation outcomes (45,70); neither of these hypotheses, however, could be verified with the data available because few studies specified whether the supervised intervention was administered individually or in groups, and none of the studies reported flexibility outcomes grouped by sex.

Eccentric training was expected to have a larger effect on joint flexibility, given the existing literature in support of this hypothesis (89); however, in the included studies that used it (1,36,66,68,87), eccentric training, restricted to the hamstrings, did not seem to show a greater effect on joint flexibility compared with the other “traditional” RT interventions, although, given the small number of studies implementing eccentric training, meta-regression could not be performed for this variable. Of the 5 studies, Potier et al. (66) and Vatovec et al. (87) showed an effect size higher (0.77 ± 0.09 and 0.82 ± 0.35, respectively) than the overall estimate, whereas the ES estimated in Junior et al. (36) and Abdel-aziem et al. (1) were slightly lower than our estimate (0.47 ± 0.05 and 0.51 ± 0.21, respectively); finally, Ribeiro-Alvares et al. (68) found an effect much lower than our pooled estimate, with a large variance because of the smaller sample size of the study (0.12 ± 0.38, *n* = 20). Furthermore, the eccentric phase of repetitions was also performed in the other “traditional” RT interventions because none of the studies reported prescribing a “concentric-only” RT protocol, so it is not possible to draw a clear line between concentric and eccentric exercise intervention.

The results of the risk of bias assessment were penalized by the lack of a preprepared analysis plan, which was only provided by one paper (8). In a similar fashion, only 4 studies reported the randomization procedure in meaningful details. This might have been a factor in determining the nonsignificance of randomization as a moderator in our meta-regression because improperly applied randomization procedures will impair the efficacy of the randomization itself.

In total, 14 studies (of the 36 included in the meta-analysis) reported dropouts from at least one group; the specific causes of dropouts where seldom reported and were mostly attributed to external reasons: for example, Fukuchi et al. (24) reported (across RT group and CG) 4 dropouts for “time constraints” and 6 for reasons unrelated to the study; Barbosa et al. (10) reported 3 dropouts for “personal reasons”; da Costa et al. (18), which had the highest number of dropouts (38 from 83 initial participants) reported, in total, 20 dropouts (18 from CG) for not appearing at evaluations, 5 for carrying out other physical activity programs, and 4 (in the RT group) for low attendance. Overall, there was a ∼6% dropout rate (93/1,469) across the included studies. The lack of intention-to-treat analysis means that the reported results can only be applied “per protocol,” which means that there is evidence of the efficacy of RT on joint flexibility only when the participants completed the assigned intervention. Still, the reported rate of dropouts was well below the threshold recommended in the 2009 Updated Method Guidelines for Systematic Reviews in the Cochrane Back Review Group (25), which improves the external validity of these results.

The present work had to be limited in scope by only including RT interventions and CG, and thus, the effect of combined intervention could not be assessed within this meta-analysis. A thorough sensitivity analysis could be conducted to assess the impact of large ES, whereas additional searches and analyses could be conducted by including mixed interventions (Flex, RT + aerobic, RT + Flex, etc.) and comparing them against isolated RT or CG using network meta-analysis techniques to rank the interventions' effect on joint flexibility.

The presence of particularly large ES (reported by the authors or estimated), both for flexibility and strength outcomes, could pose a problem to the validity of this analysis. Sensitivity analysis, performed by removing all ES >3, however, confirms our results, with the pooled estimate differing from the full model by just ∼ −0.015.

The corresponding authors were asked to verify these results because there is evidence of incorrect ES reporting in a few sport science trials (34), but, at the present moment, and despite our numerous attempts to reach out to them, the large majority of the authors could not be contacted.

The first possibility to explain these results would be to attribute it to chance or to a reporting error in the original papers. Another possible explanation is that the distribution of these ES or the random effects is heavy tailed to the right. This would require slight adjustments to the model used for estimating the overall effect, by using a different distribution using the maximum likelihood method.

In respect to the work of Afonso et al. (3), who compared the effect of resistance training vs a stretching intervention, and found a similar effect on flexibility for both training modalities (slightly favoring stretching, ES = −0.22; 95% CI = −0.55 to 0.12; *p* = 0.206), our work focused on case-control designs (i.e., comparing RT to control group), and thus is not directly comparable. Still, the scope of the analyses can be qualitatively compared: for this work, a much larger number of items (18,355 vs 121) were screened, and the initial, intercept-only model encompassed more than thrice the studies (36 vs 11). Furthermore, the present search was restricted to only include healthy, adult participants, which reduced the possible influence of confounding factors such as the effect of illnesses or biological maturation on the musculoskeletal system.

Our results are in general accordance with the recent review of Alizadeh et al. (5), although we chose to exclude studies where Pilates was the main intervention because it generally also involves a flexibility training component, and we would not consider it a form of resistance training comparable with training with free weights or machines. Furthermore, our systematic review and meta-analysis has a number of methodological upsides: we computed a multilevel meta-analytic model, which takes into account the dependency of ESs nested at the study level. We also ran a number of diagnostic tests to assess the validity of our model, and used the methods recommended by the Cochrane to assess risk of bias, namely RoB2 and ROBINS-I; conversely, Alizadeh et al., apparently only assessed publication bias, by use of funnel plots and Egger's regression test, and assessed methodological quality of their included trials using the PEDro scale, which has received some criticisms in regard to its psychometric properties (4).

The effect of resistance training on flexibility should be explored further, and we suggest that future studies could compare, for example, the effect on flexibility of RT performed at long muscle lengths, or through the full ROM of the involved joint(s), against exercises that do not place the muscle in an elongated position, or with limited ROM. In addition, given the significant effect of training intensity, one suggestion for future studies would be to directly assess the effect of low vs high intensity resistance training on flexibility.

The limited reporting of covariates, especially regarding resistance training protocols, restricted the possibility to accurately estimate dose-response relationships with joint flexibility gains, and the substantial heterogeneity, with PIs crossing the null effect means that different RT protocols may have different true effects on joint flexibility and prevents us from concluding that RT has a positive effect on joint flexibility.

**Practical Applications**

The possibility of improving joint flexibility through resistance training could open to the possibility of more time-efficient and enjoyable training routines for different age brackets and levels of training. Given the large strength improvements in the first months of resistance training, this modality of training may be favored when considering time and resources allocation constraints. Resistance training is already included in most international guidelines for physical activity because of its effects on muscle functionality, bone strength, as well as health-related quality of life in older adults (30,95); therefore, the perspective of improving joint flexibility at the same time could further strengthen the argument for its inclusion in most training routines. Still, the results of this work should not be interpreted as a basis to discard stretching exercise routines, which are still important as a safe and easy to administer complement in training routines for healthy persons. Moreover, the efficacy of resistance training alone remains to be compared with combined interventions and stretching exercises, so the possibility of further benefits compared with resistance training alone cannot be excluded at the present moment.

## Supplementary Material

SUPPLEMENTARY MATERIAL
