# Supplementary material for: The Influence of Resistance Training on Joint Flexibility in Healthy Adults: A Systematic Review, Meta-analysis, and Meta-regression
Source: J Strength Cond Res. 2024 Dec 31;39(3):386–97. doi: 10.1519/JSC.0000000000005000 (PMC11841725; doi:10.1519/JSC.0000000000005000)
Supplement: SUPPLEMENTARY MATERIAL [file jscr-39-0386-s001.pdf]

Search Strategy

1. "Range of Motion, Articular"[Mesh]
2. "range of motion, articular"
3. "range of motion"
4. "goniometer"
5. "goniometry"
6. "inclinometer"
7. "inclinometry"
8. ("stretching" AND "perception")
9. extensib*
10. muscle pliab*
11. "passive torque"
12. "passive rom"
13. "joint angle"
14. "joint excursion"
15. "joint flexibility"
16. "joint mobility"
17. "tendon lengthen*"
18. "muscle lengthen*"
19. "muscle length"
20. "stretch sensitivity"
21. "stretching technique"
22. "tight hamstrings"
23. "Muscle Stretching Exercises"[Mesh]
24. "Muscle Stretching Exercises"
25. "proprioceptive neuromuscular facilitation"
26. "active stretching"

27. "dynamic stretching"
28. chronic stretching
29. "passive stretching"
30. "static stretching"
31. "stretch training"
32. "stretching program"
33. "flexibility training"
34. "flexibility program"
35. "joint flexib*"
36. "muscle flexib*"
37. "muscle elastic*"
38. "active knee extension test"
39. "back scratch"
40. "passive knee extension test"
41. "straight leg raise"
42. "side bending"
43. "sit and reach"
44. "thomas test")
45. OR/ 1-44
46. "resistance training"[Mesh]
47. "resistance training"
48. "gymnastics"[Mesh]
49. "gymnastics"
50. "calisthenics"
51. "strength training"
52. ("strength" AND "workout")
53. "strength development"
54. "strengthening program"

55. powerlift*
56. "weight lifting"
57. "weightlifting"
58. "weight training"
59. "time under tension"
60. "theraband"
61. "elastic bands"
62. "resistance exercise"
63. "body weight exercise"
64. "bodyweight exercise"
65. "free weight exercise"
66. "power training"
67. "resistive training"
68. "isometric exercise"
69. "isotonic exercise"
70. "eccentric exercise"
71. "eccentric overload"
72. "peak muscle strength"
73. "1RM"
74. "maximal strength"
75. "maximum strength"
76. "muscle strengthen*"
77. OR/ 45-76
78. 45 AND 77
79. "cross-sectional area"
80. "fat-free mass"
81. "lean body mass"
82. "muscle hypertrophy"

83. "muscle gain"

84. "muscle growth"

85. "muscle size"

86. "muscle thickness"

87. "skeletal muscle mass"

88. OR/ 79-87

89. 77 OR 88

90. 45 AND 89
