# Supplementary material for: The Influence of Resistance Training on Joint Flexibility in Healthy Adults: A Systematic Review, Meta-analysis, and Meta-regression
Source: J Strength Cond Res. 2024 Dec 31;39(3):386–97. doi: 10.1519/JSC.0000000000005000 (PMC11841725; doi:10.1519/JSC.0000000000005000)
Supplement: SUPPLEMENTARY MATERIAL [file jscr-39-0386-s002.pdf]

*Table 1b - Study summary*

| Study | Participants | Groups interventions and numerosity | Volume and intensity | Frequency x duration | Main muscle groups | Outcomes |
|---|---|---|---|---|---|---|
| **Balogun et al, 1992** | *Desc*: Healthy male college students<br>*n*: 34<br>*Age (y)*: 23.1 ± 3.1<br>*Activity lvl*: Unreported | RT-1: Supine exercise group, glute bridge (11)<br>RT-2: Prone exercise group, back extension (12)<br>CG: no exercise (11) | RT-1, RT-2: 1 set of 20 reps held for 5s | 3 times x 6weeks | RT-1: gluteus maximus<br>RT-2: Erector spinae | *Flexibility*: Standing trunk extension (cm)<br>*Strength*: Standing trunk extension dynamometry (kg)<br>*Other*: - |
| **Wood et al, 2001** | *Desc*: Healthy older adults<br>*n*: 36<br>*Age (y)*: 69.1±5.3 (CVT), 69.8±6.0 (RT), 66.1±5.5 (CV+RT), 68.0±5.4 (CG)<br>*Activity lvl*: Moderately active | CVT: Cardiovascular training on treadmill and cycle-ergometers (10)<br>RT: Strength training program with free weight and machines (11)<br>CV+RT: cardiovascular and strength training (9)<br>CG: Maintain usual habits (6) | CVT: 45 min @60-70% HRMax, 11-13 RPE<br>RT: 2x8-12 @75-85% 1RM | 3 times x 12 weeks | CVT: Lower body<br>RT: Major muscle groups | *Flexibility*: Sit and reach (inches)<br>*Strength*: 5RM tests: Leg-extension, Leg-Curl, Seated Row, Chest-Press, Lateral Raise, Seated Dip, Biceps-Curl (kg)<br>*Other*: AAHPERD assessment of functional fitness, Submaximal cardiorespiratory test |
| **Barbosa et al, 2002** | *Desc*: Older women<br>*n*: 19<br>*Age (y)*: 68.91±5.43 (RT), 65.13±4.09 (CG)<br>*Activity lvl*: Sedentary | RT: Strength training program with free weight and machines (11)<br>CG: Maintain usual habits (8) | RT: 3-5 x 6-15; 1 set at low-moderate intensity, 2 to momentary failure | 3 times x 10 weeks | RT: Major muscle groups | *Flexibility*: Sit and reach (cm)<br>*Strength*: -<br>*Other*: - |

| | | | | | | |
|---|---|---|---|---|---|---|
| **Fatouros et al, 2002** | *Desc*: Aged men<br>*n*: 32<br>*Age (y)*: 70.5±5.9 (CG), 71.8±2.5 (CVT), 70.3±2.3 (RT), 69.8±1.9 (CV+RT)<br>*Activity lvl*: Sedentary | CVT: Cardiovascular training on treadmill (8)<br>RT: Strength training program on universal RT machines (8)<br>CV+RT: cardiovascular and strength training (8)<br>CG: no training (8) | CVT: 42 min, "Training intensity was set at […] 80%"<br>RT: 4x8 @80% 1RM | 3 times x 16 weeks | CVT: Lower body<br>RT: Major muscle groups | *Flexibility*: Modified sit and reach (cm); Goniometry ROM of hip flexion, extension, abduction, adduction, shoulder extension, flexion, adduction, knee extension/flexion and elbow extension/flexion (degrees)<br>*Strength*: knee flexion and extension peak torque at 60 deg/s and 180 deg/s (Nm); chest press and leg press 1RM (kg)<br>*Other*: Aerobic capacity, anthropometric variables |
| **Rogers et al, 2002** | *Desc*: Aged African-American women<br>*n*: 22<br>*Age (y)*: 74.8 ± 8.8 (RT), 74.7 ± 4.5 (CG)<br>*Activity lvl*: Sedentary | RT: Strength training program with free weight and elastic bands (16)<br>CG: Maintain usual habits (6) | RT: 3 x 8-15 | 3 times x 4 weeks | RT: Major muscle groups | *Flexibility*: Back scratch (cm), Modified sit and reach (cm)<br>Strength: Handgrip (kg);<br>*Other*: Timed up and go (s), 30s arm curl, 5 times sit to stand (s), anthropometric variables |
| **Cyrino et al, 2004** | *Desc*: Healthy young men<br>*n*: 16<br>*Age (y)*: 23.0 ± 2.1<br>*Activity lvl*: Sedentary or moderately active | RT: Circuit strength training (8)<br>CG: No involvement in regular exercise (8) | RT: 3x8-12 to momentary failure | 3 times x 10 weeks | RT: Major muscle groups | *Flexibility*: Fleximetry ROM of shoulder extension, flexion, elbow extension, flexion, hip extension, flexion, trunk lateral flexion, flexion, extension, and knee flexion (degrees)<br>*Strength*: -<br>*Other*: - |
| **Kalapotharakos et al, 2005** | *Desc*: Older, independent participants<br>*n*: 33<br>*Age (y)*: 64.64±5.14 (HRT), 65.75±4.25 (MRT), 64.4±3.37 | HRT: high intensity strength training on universal gym machines (11)<br>MRT: medium intensity strength training on universal gym machines | HRT: 3x8 @80% 1RM<br>MRT: 3x15 @60% 1RM | 3 times x 12 weeks | HRT, MRT: Major muscle groups | *Flexibility*: Sit and reach (cm)<br>*Strength*: 1RM lower body strength (sum of knee flexion and extension 1RM; kg)<br>*Other*: Walking speed (m/s), chair rising (s), Stair climbing (s) |

| | | | | | | |
|---|---|---|---|---|---|---|
| | (CG)<br>*Activity lvl*: Sedentary | (12)<br>CG: did not exercise (10) | | | | |
| **Nobrega et al, 2005** | *Desc*: Young healthy participants<br>*n*: 43<br>*Age (y)*: 21 ± 4<br>*Activity lvl*: Sedentary | FLEX: Self-administered static stretching (11)<br>RT: Strength training program with free weight and machines (13)<br>FLEX+RT: stretching and strength training (9)<br>CG: participants remained sedentary (10) | FLEX: 3x30" hold per exercise, maximal amplitude before pain<br>RT: 3x8-12 @60% 1RM | 2 times x 12 weeks | FLEX: joints of upper and lower limbs, hip and trunk<br>RT: Major muscle groups | *Flexibility*: Flexitest score<br>*Strength*: Handgrip, bench press and leg press 1RM (kg)<br>*Other*: anthropometric variables |
| **Fatouros et al, 2006** | *Desc*: Aged Caucasian males<br>*n*: 50<br>*Age (y)*: 69.8 ± 5.1 (LRT), 71.1 ± 3.6 (MRT), 69.7 ± 3.8 (HRT), 70.8 ± 2.8 (CG)<br>*Activity lvl*: Sedentary | LRT: Low intensity strength training program with free weight and machines (14)<br>MRT: Medium intensity strength training program with free weight and machines (12)<br>HRT: High intensity strength training program with free weight and machines (14)<br>CG: Maintain usual habits (10) | LRT: 3 x 14.6 ± 0.8 @ 46.9% ± 2.3% 1RM<br>MRT: 3 x 10.1 ± 1.1 @ 62.8 ± 3.0% 1RM<br>HRT: 3 x 7.9 ± 0.4 @ 82.2 ± 1.8% 1RM | 3 times x 24 weeks | LRT, MRT, HRT: Major muscle groups | *Flexibility*: Goniometry ROM of hip flexion and extension, Shoulder flexion and extension, knee flexion, elbow flexion (degrees), modified sit and reach (cm)<br>*Strength*: Chest press and leg press 1RM<br>*Other*: Aerobic capacity |

| | | | | | | |
|---|---|---|---|---|---|---|
| **Simons et al, 2006** | *Desc*: Aged Caucasian healthy adults<br>*n*: 64<br>*Age (y)*: 83.5 ± 6.2<br>*Activity lvl*: Sedentary | RT: Six full-ROM strength exercises using machines (21).<br>WK: Walking indoors and outdoors (18).<br>CG: Lectures on health, training and nutrition (21) | RT: 3-5 x 10 @75% 1RM | 2 times x 16 weeks | Major muscle groups | *Flexibility*: Goniometry ROM of hip flexion, Shoulder flexion and abduction (degrees), sit and reach (cm)<br>*Strength*: Leg extension, leg curl, leg press, lat pull-down, upper back machine, chest press machine 1RM. |
| **Moreira Junior et al, 2007** | *Desc*: Male physical education students<br>*n*: 22<br>*Age (y)*: 23.2±2.4 (EG), 24.2±5.2 (CG)<br>*Activity lvl*: - | Experimental group (13) trained each leg differently:<br>ERT: Eccentric leg curl training on one leg<br>ERT+FLEX: Eccentric leg curl training and<br>passive static stretching on the other leg<br>CG: maintain usual habits (9) | ERT: 3x10-12 @70% 1RM<br>FLEX: 4 sets of 20" | ERT: 3 times x 6 weeks<br>FLEX: 2 times x 6 weeks | ERT, ERT+FLEX: Hamstrings | *Flexibility*: Hamstring passive ROM (degrees)<br>*Strength*: Hamstrings 1RM<br>*Other*: - |
| **Peixoto et al, 2007** | *Desc: Male physical education students*<br>*n: 22*<br>*Age (y)*: 22.1±1.4 (EG), 24.2±5.2 (CG)<br>*Activity lvl*: no RT or stretching in the past 3 months | Experimental group (12) trained each leg differently:<br>RT: Leg curl training on one leg<br>RT+FLEX: Leg curl training and<br>passive static stretching on the other leg<br>CG: maintain usual habits (10) | RT: 3x10-12 @70% 1RM<br>FLEX: 4 sets of 20" | 2 times x 6 weeks | RT, RT+FLEX: Hamstrings | *Flexibility*: Hamstring passive ROM (degrees)<br>*Strength*: Hamstrings 1RM<br>*Other*: Hamstrings stiffness |

| | | | | | | |
|---|---|---|---|---|---|---|
| **Monteiro et al, 2008** | *Desc*: Healthy women<br>*n*: 20<br>*Age (y)*: 37±1.7 (RT), 36.9±1.2 (CG)<br>*Activity lvl*: Sedentary | RT: Circuit strength training (10)<br>CG: Not described (10) | RT: 3x8-12 @8-12RM | 3 times x 10 weeks | RT: Major muscle groups | *Flexibility*: Goniometry ROM of shoulder flexion, extension, horizontal adduction and abduction, elbow flexion, hip flexion and extension, knee flexion and extension, trunk flexion and extension (degrees)<br>*Strength*: 10RM test for bench press, Smith machine squat, lat pulldown, leg press, incline bench press, hack machine (kg)<br>*Other*: - |
| **Kasser et al, 2009** | *Desc*: Healthy adults<br>*n*: 27<br>*Age (y)*: 24.2 (SS), 23.8 (RT), 24.7 (CG)<br>*Activity lvl*: - | SS: passive static stretching (9)<br>RT: free weight strength training (9)<br>CG: Maintain usual habits (9) | SS: 3x30s holds<br>RT: 3x10 | 5 times x 6 weeks | SS: gastrocnemius<br>RT: Tibialis anterior | *Flexibility*: Goniometry of active dorsiflexion ROM (degrees)<br>*Strength*: -<br>*Other*: - |
| **Potier et al, 2009** | *Desc*: Healthy adults<br>*n*: 22<br>*Age (y)*: 27±0.8 (ERT), 29.6±1.2 (CG)<br>*Activity lvl*: No regular training | ERT: eccentric leg curl training (11)<br>CG: Not described (11) | ERT: 3x8 ecc reps @1RM | 3 times x 8 weeks | ERT: Hamstrings | *Flexibility*: Passive knee extension test (degrees)<br>*Strength*: Eccentric hamstrings 1RM (kg)<br>*Other*: hamstrings Fascicle length (mm), pennation angle (degrees) |
| **Santos et al, 2010** | *Desc*: Young sedentary women<br>*n*: 24<br>*Age (y)*: 26.8±1.6 (PRT), 24±2.3 (ART), 25.4±2.4 (CG)<br>*Activity lvl*: Sedentary | PRT: Strength training with paired agonist/antagonist exercises (8)<br>ART: Strength training with paired upper/lower body exercise (8)<br>CG: Not described (8) | PRT: 3x10-12 to concentric failure<br>ART: 3x10-12 to concentric failure | 3 times x 8 weeks | PRT, ART: Major muscle groups | *Flexibility*: Goniometry ROM of shoulder flexion, extension, horizontal adduction and abduction, trunk flexion and extension (degrees)<br>*Strength*: Bench press 1RM (kg)<br>*Other*: - |

| | | | | | | |
|---|---|---|---|---|---|---|
| **Junior et al, 2011** | *Desc*: Healthy young men<br>*n*: 60<br>*Age (y)*: -<br>*Activity lvl*: Recreationally active | RT1: Strength training with machines and free weights, one set (20)<br>RT3: Strength training with machines and free weights, three sets (20)<br>CG: didn't participate in strength training (20) | RT1: 1x8-12 to concentric failure<br>RT3: 3x8-12 to concentric failure | 3 times x 10 weeks | RT1, RT3: Major muscle groups | *Flexibility*: Sit and reach (cm)<br>Strength: Bench press and leg press 5RM (kg)<br>*Strength*: -<br>*Other*: - |
| **Kim et al, 2011** | *Desc*: Healthy, college aged women<br>*n*: 35<br>*Age (y)*: 20.8 ± 0.8 (RT), 19.5 ± 0.3 (SRT), 21.5 ± 0.8 (CG)<br>*Activity lvl*: No structured exercise in the last 6 months | RT: Traditional resistance training with machines and free weights (13)<br>SRT: Superslow resistance training with machines and free weights (14)<br>CG: Maintain usual habits (8) | RT: 3x8 @80% 1RM<br>SRT: 1 set to fatigue, 10s concentric and eccentric phases @50%1RM | RT: 3 times x 4 weeks<br>SRT: 2 times x 4 weeks | RT, SRT: Major muscle groups | *Flexibility*: Sit and reach (cm)<br>*Strength*: Shoulder press, chest press, leg press, low row and lat pulldown 1RM tests (kg)<br>*Other*: Maximum aerobic capacity |
| **Morton et al, 2011** | *Desc*: Young, healthy university students<br>*n*: 36<br>*Age (y)*: 21.92±3.64<br>*Activity lvl*: Inactive (CG) | RT: Strength training with machines and free weights (12)<br>SS: Static stretching program (12)<br>CG: Convenience control group (12) | RT: 4 sets per exercise<br>SS: 1-3 sets per 20-30s | 3 times x 5 weeks | RT, SS: Major muscle groups | *Flexibility*: Goniometry ROM of knee extension, hip flexion and extension (degrees), arm lift test (inches)<br>*Strength*: knee extension and flexion peak torque at 180 deg/s (Nm)<br>*Other*: - |
| **Simao et al, 2011** | *Desc*: Untrained, adult women<br>*n*: 80<br>*Age (y)*: 35±2.0 (RT), 34±1.2 (SS), 35± 1.8 (RT+FLEX), 34± 2.1 (CG)<br>*Activity lvl*: Sedentary | RT: Strength training with machines and free weights (20)<br>SS: Static stretching program (20)<br>RT+FLEX: static stretching and strength training in each session | RT: 3x8-12<br>SS: 4x15-60s | 3 times x 16 weeks | RT, SS, RT+FLEX: Major muscle groups | *Flexibility*: Sit and reach (cm)<br>Strength: Leg press and bench press 10RM (kg)<br>*Strength*: -<br>*Other*: - |

| | | | | | | |
|---|---|---|---|---|---|---|
| | | (20)<br>CG: Not described (20) | | | | |
| **da Costa et al, 2013** | *Desc*: Aged healthy adults<br>*n*: 45<br>*Age (y)*: 67.5±2<br>*Activity lvl*: - | RT: Lower body strength training with machines and free weights (13)<br>FLEX: Lower body active stretching program (10)<br>RT+FLEX: Stretching and strength training in each session (9)<br>CG: received healthy lifestyle advice (13) | RT: 3x8 @75% 10RM<br>FLEX: 4x60s stretching | 2 times x 12 weeks | RT, FLEX, RT+FLEX: Lower body muscles | *Flexibility*: Fleximetry ROM of bilateral knee flexion, hip flexion and extension, dorsiflexion and plantarflexion (degrees)<br>*Strength*: -<br>*Other*: - |
| **Takeshima et al, 2013** | *Desc*: Community-dwelling se*niors*<br>*n*: 65<br>*Age (y)*:<br>NW: 70.1 ±5.3;<br>CW: 68.0 ±4.9<br>RES: 68.0 ±5.4;<br>CON: 70 ± 7<br>*Activity lvl*: Sedentary | NW: Nordic walking (17)<br>CW: Walking (16)<br>RT: Strength training with elastic bands (9)<br>CG: - (17) | RT: 50-60 min @11-13 up to 15-17 RPE | 2 times x 12 weeks | All major muscles groups | *Flexibility*: Back scratch, chair sit and reach (cm)<br>*Strength*: -<br>*Other*: - |
| **Yaprak et al, 2013** | *Desc*: University students<br>*n*: 73<br>*Age (y)*:<br>EX: 18.17±0.61;<br>CG: 20.39±1.88<br>*Activity lvl*: - | EX: Back extension exercise (35)<br>CG: No activity (38) | EX: 2 x 25 @80% of max reps | 4 times x 10 weeks | Back extensors muscles | *Flexibility*: Goniometry ROM of lumbar and thoracic flexion and extension (degrees)<br>*Strength*: Isometric back extension strength (Kg of force)<br>*Other*: - |

| | | | | | | |
|---|---|---|---|---|---|---|
| **Fukuchi et al, 2016** | *Desc*: Healthy, recreational runners<br>*n*: 91<br>*Age (y)*: 59.8±4.7 (RT), 59.8±4.0 (FLEX), 59.9±3.6 (CG)<br>*Activity lvl*: Recreationally active | RT: Home based, lower body strength training (36)<br>FLEX: Home based, lower body static stretching program (34)<br>CG: advised to avoid exercise programs (35) | RT: 3x15<br>FLEX: 4x15-30s, to the point of mild discomfort | 6 times x 8 weeks | RT, FLEX: Lower body muscles | *Flexibility*: Goniometry ROM of hip adduction, extension, internal rotation, ankle dorsiflexion (degrees)<br>*Strength*: MVIC of hip abductors and extensors, ankle plantar flexors (%BW)<br>*Other*: running biomechanical measures |
| **Kim et al, 2017** | *Desc*: Older healthy women<br>*n*: 21<br>*Age (y)*: 76.10±3.85 (RT), 76.40±3.27 (CG)<br>*Activity lvl*: Inactive in the past 3 months | RT: Free weights and bodyweight strength training (11)<br>CG: Maintain daily activities, no exercise (10) | RT: 2-3 sets @ RPE 11-13 | 3 times x 24 weeks | RT: Major muscle groups | *Flexibility*: Back scratch, sit and reach (cm)<br>*Strength*: 30s arm curl, 30s chair stand<br>*Other*: Senior fitness test, hippocampus volume ($mm^3$) |
| **Leite et al, 2017** | *Desc*: Men from the Brazilian Navy School of Lieutenants<br>*n*: 47<br>*Age (y)*: 24±1<br>*Activity lvl*: military trained, no RT experience | RT1: Strength training with machines and free weights, one set (12)<br>RT3: Strength training with machines and free weights, three sets (13)<br>RT5: Strength training with machines and free weights, five sets (13)<br>CG: didn't participate in strength training (9) | RT1: 1x8-12 to volitional failure<br>RT3: 3x8-12 to volitional failure<br>RT5: 5x8-12 to volitional failure | 3 times x 24 weeks | RT1, RT3, RT5: Major muscle groups | *Flexibility*: Sit and reach (cm), goniometry ROM of shoulder flexion, extension, horizontal adduction and abduction, elbow flexion, hip flexion and extension, knee flexion, trunk flexion and extension (degrees)<br>*Strength*: -<br>*Other*: - |
| **Ribeiro-alvares et al, 2017** | *Desc*: Healthy young students<br>*n*: 20<br>*Age (y):*<br>TG: 23.7 ± 3.3;<br>CG: 26.0 ± 2.7 | RT: Nordic hamstrings exercise (10)<br>CG: - (10) | RT: 3x6-10 | 2 times x 4 weeks | RT: Hamstrings | *Flexibility*: Sit and reach (cm)<br>Strength: Isokinetic dynamometry: isometric, concentric, eccentric peak torque<br>*Strength*: -<br>*Other*: - |

| | | | | | | |
|---|---|---|---|---|---|---|
| | *Activity lvl*: Moderately active | | | | | |
| **Smith et al, 2017** | *Desc*: Healthy, older adults<br>*n*: 16<br>*Age (y)*: 56.1±4.5 (RT), 59.0±5.9 (CG)<br>*Activity lvl*: Sedentary | RT: Home based strength training (7)<br>CG: Remained sedentary and self-reported daily activities (9) | RT: 2-3x15-20 @6-8 on the OMNI scale | 3 times x 8 weeks | RT: Major muscle groups | *Flexibility*: Sit and reach (cm)<br>Strength: Handgrip (kg), knee push-up test and 1-min squat test<br>*Other*: Microvascular assessment, anthropometric assessment, cardiopulmonary exercise assessment, SF-36, functional reach test (cm) |
| **Fritz et al, 2018** | *Desc*: Older, overweight women<br>*n*: 75<br>*Age (y)*: 69.2 ± 1.06 (ET); 70.43 ± 0.97 (EB); 67.2 ± 1.06 (CG)<br>*Activity lvl*: Sedentary | ET: Group resistance training using elastic tubes (25)<br>EB: Group resistance training using elastic bands (25)<br>CG: Maintain habits (25) | ET, EB: 3-4x10 @7-9 on the OMNI scale | 2 times x 8 weeks | ET, EB: Upper and lower limbs | *Flexibility*: Back scratch (cm), sit and reach (cm)<br>*Strength*: Isometric strength test of upright row, squat and trunk extension (kg)<br>*Other*: Anthropometric measurements, senior fitness test |
| **Abdel-Aziem et al, 2018** | *Desc*: physical therapy students<br>*n*: 60<br>*Age (y)*: 22.18±2.85 (URT), 21.73±2.67 (TRT), 21.08±2.39 (CG)<br>*Activity lvl*: trained and untrained individuals, divided in different groups | ERT: Divided between untrained individuals (URT, 20) and trained individuals (TRT, 20); eccentric hamstrings training<br>CG: Untrained individuals, did not exercise (20) | ERT: 6 reps of 5s each @40% 1RM | 5 times x 6 weeks | ERT: Hamstrings | *Flexibility*: Goniometry ROM of knee extension (degree)<br>*Strength*: Hamstring eccentric and concentric peak torque at 60 deg/s and 120 deg/s (Nm)<br>*Other*: - |

| | | | | | | |
|---|---|---|---|---|---|---|
| **Solà-Serrabou et al, 2019** | *Desc*: independent elderly individuals<br>*n*: 30<br>*Age (y)*: 71.9±5.0 (RT), 74.8±6.1 (CG)<br>*Activity lvl*: Sedentary | RT: Bodyweight and elastic bands-based resistance training (18)<br>CG: Not described (12) | RT: 1-3x8-15 @5-6/10 Borg scale | 2 times x 24 weeks | RT: Lower body muscles | *Flexibility*: Modified sit and reach, back scratch (cm)<br>*Strength*: chair stand test, squat jump (cm), countermovement jump (cm)<br>*Other*: 2-min step test, Yesavage depression scale, SF-12 |
| **Baker et al, 2020** | *Desc*: Older adults<br>*n*: 46<br>*Age (y)*: 68.2±6.7 (RT), 68.6±8.7 (WALK), 67.6±6.9 (CG)<br>*Activity lvl*: Sedentary | RT: "Stay healthy, stay strong" program, dumbbells and bodyweight resistance training (15)<br>WALK: Walking (17)<br>CG: refrained from structured exercise programs (14) | RT: 2x6-10 not to failure<br>WALK: 50 mins at self-selected pace | 2 times x 8 weeks | RT: Major muscle groups<br>WALK: Lower body | *Flexibility*: Back scratch, modified sit and reach (cm)<br>*Strength*: Handgrip (kg), 30s sit to stand<br>*Other*: 10m walk test (m/s), 8ft timed up and go (s), CDC four-stage balance test, 10s quiet standing, PSQI, SAGE scores |
| **Piraua et al, 2020** | *Desc*: Older women<br>*n*: 58<br>*Age (y)*: 68.11 ± 3.89 (RT); 66.94 ± 5.65 (URT); 67.50 ± 4.75 (CG)<br>*Activity lvl*: Sedentary (last 6 months) | RT: Strength training with machines and free weights (22)<br>URT: Strength training + unstable surfaces (22)<br>CG: Encouraged to maintain habits (14) | RT, URT: 2-5 x 7-12, intensity not reported | 3 times x 24 weeks | RT, URT: Major muscle groups | *Flexibility*: Sit and reach (cm)<br>*Strength*: Handgrip (kg)<br>*Other*: - |
| **Elsangedy et al, 2021** | *Desc*: Older women<br>*n*: 32<br>*Age (y)*: 65.7±3.3 (SSRT); 66.3±2.8<br>*Activity lvl*: Sedentary | SSRT: Self-selected resistance training (16)<br>CG: Board games and activities at the same frequency (16) | SSRT: 3 x 15 @ self-selected intensity | 3 times x 12 weeks | SSRT: Major muscle groups | *Flexibility*: Chair sit and reach, back scratch (cm), passive knee extension test, single and bilateral hip flexors flexibility (degrees)<br>*Strength*: 1RM test of bench press, leg press, lateral pulldown, knee extension, lateral shoulder raise, knee curl, biceps curl, and triceps pushdown (kg), isokinetic peak |

| | | | | | | |
|---|---|---|---|---|---|---|
| | | | | | | torque tests of leg flexors and extensors (Nm), handgrip (kg)<br>*Other*: - |
| **Vatovec et al, 2021** | *Desc*: Physically active volunteers<br>*n*: 40<br>*Age (y)*: 24.2 ± 2.1 (TG); 23.0 ± 2.8 (CG)<br>*Activity lvl*: Physically active at least 3 times per week | TG: Eccentric hamstring exercise (20)<br>CG: Maintain habits (20) | TG: 2-3 sets per exercise @8 RPE | 2 times x 6 weeks | TG: Nordic hamstring and glider exercises | *Flexibility*: Passive straight leg raise (degrees)<br>*Strength*: -<br>*Other*: - |
| **Versic et al, 2021** | *Desc*: Healthy young women<br>*n*: 57<br>*Age (y)*: 23.9 ± 3.08<br>*Activity lvl*: No previous expertise in exercise | RT: Supervised circuit weight training (19)<br>ET: Endurance training (18)<br>CG: Not involved (20) | RT: 3 sets to failure<br>ET: 45-60 minutes below anaerobic threshold | 3 times x 8 weeks | RT: Major muscle groups<br>ET: treadmill running | *Flexibility*: Sit and reach, shoulder circumduction test (cm)<br>*Strength*: Handgrip (kg)<br>*Other*: - |

1, 5, 8, 10RM: 1, 5, 8, 10 repetition maximum; CG: Control group; CVT: Cardiovascular training; ERT: Eccentric resistance training; FLEX: Flexibility training; MVIC: Maximum voluntary isometric contraction; OMNI: RPE scale for resistance exercise; ROM: Range of motion; RPE: Rate of perceived effort; RT: Resistance/strength training; SS: Static stretching; VAS: Visual analogue scale.

Ages are reported as Mean ± S.D. in Years; Volume is described as Sets x Repetitions @ intensity
