# Supplementary material for: The Influence of Resistance Training on Joint Flexibility in Healthy Adults: A Systematic Review, Meta-analysis, and Meta-regression
Source: J Strength Cond Res. 2024 Dec 31;39(3):386–97. doi: 10.1519/JSC.0000000000005000 (PMC11841725; doi:10.1519/JSC.0000000000005000)
Supplement: SUPPLEMENTARY MATERIAL [file jscr-39-0386-s003.pdf]

Table 1c - RoB2 summary table

Study ID	Outcome	Weight	D1	D2	D3	D4	D5	Overall
1992_Balogun	back extension rom	1	-	!	+	-	!	-
1992_Balogun	back extension strength	1	-	!	+	-	!	-
2001_Wood	sit and reach	1	!	!	-	-	!	-
2001_Wood	5rm of leg extension	1	!	!	-	-	!	-
2001_Wood	5rm of leg curl	1	!	!	-	-	!	-
2001_Wood	5rm of low row	1	!	!	-	-	!	-
2001_Wood	5rm of chest press	1	!	!	-	-	!	-
2001_Wood	5rm of lateral raises	1	!	!	-	-	!	-
2001_Wood	5rm of seated dip	1	!	!	-	-	!	-
2001_Wood	5rm of biceps curl	1	!	!	-	-	!	-
2002_Fatouros	knee peak torque at 60°/s	1	!	!	-	+	!	-
2002_Fatouros	knee peak torque at 180°/s	1	!	!	-	+	!	-
2002_Fatouros	1RM of chest press	1	!	!	-	+	!	-
2002_Fatouros	1RM of leg press	1	!	!	-	+	!	-
2002_Fatouros	modified sit and reach	1	!	!	-	!	!	-
2002_Fatouros	goniometric assessment of hip flexion	1	!	!	-	!	!	-
2002_Fatouros	goniometric assessment of hip extension	1	!	!	-	!	!	-
2002_Fatouros	goniometric assessment of hip abduction	1	!	!	-	!	!	-
2002_Fatouros	goniometric assessment of hip adduction	1	!	!	-	!	!	-
2002_Fatouros	goniometric assessment ofshoulder extension	1	!	!	-	!	!	-
2002_Fatouros	goniometric assessment of shoulder flexion	1	!	!	-	!	!	-
2002_Fatouros	goniometric assessment of shoulder adduction	1	!	!	-	!	!	-
2002_Fatouros	goniometric assessment of knee flexion/extension	1	!	!	-	!	!	-
2002_Fatouros	goniometric assessment of elbow flexion extension	1	!	!	-	!	!	-
2004_Cyrino	active shoulder flexion R	1	!	+	+	+	!	!
2004_Cyrino	active shoulder flexion L	1	!	+	+	+	!	!

2004_Cyrino	active shoulder extension R	1	!	+	+	+	!	!
2004_Cyrino	active shoulder extension L	1	!	+	+	+	!	!
2004_Cyrino	active elbow flexion R	1	!	+	+	+	!	!
2004_Cyrino	active elbow flexion L	1	!	+	+	+	!	!
2004_Cyrino	active elbow extension R	1	!	+	+	+	!	!
2004_Cyrino	active elbow extension L	1	!	+	+	+	!	!
2004_Cyrino	active hip flexion R	1	!	+	+	+	!	!
2004_Cyrino	active hip flexion L	1	!	+	+	+	!	!
2004_Cyrino	active hip extension R	1	!	+	+	+	!	!
2004_Cyrino	active hip extension L	1	!	+	+	+	!	!
2004_Cyrino	active trunk extension	1	!	+	+	+	!	!
2004_Cyrino	active trunk flexion	1	!	+	+	+	!	!
2004_Cyrino	active trunk side flexion R	1	!	+	+	+	!	!
2004_Cyrino	active trunk side flexion L	1	!	+	+	+	!	!
2004_Cyrino	active knee flexion R	1	!	+	+	+	!	!
2004_Cyrino	active knee flexion L	1	!	+	+	+	!	!
2005_Kalapotharakos	knee extension + knee flexion 1RM	1	!	!	+	+	!	!
2005_Kalapotharakos	sit and reach	1	!	!	+	+	!	!
2006_Fatouros	chest press 1RM	1	!	+	+	-	!	-
2006_Fatouros	leg press 1RM	1	!	+	+	-	!	-
2006_Fatouros	modified sit and reach	1	!	+	+	-	!	-
2006_Fatouros	goniometric assessment of hip flexion	1	!	+	+	-	!	-
2006_Fatouros	goniometric assessment of hip extension	1	!	+	+	-	!	-
2006_Fatouros	goniometric assessment of shoulder flexion	1	!	+	+	-	!	-
2006_Fatouros	goniometric assessment of shoulder extension	1	!	+	+	-	!	-
2006_Fatouros	goniometric assessment of knee flexion	1	!	+	+	-	!	-
2006_Fatouros	goniometric assessment of elbow flexion	1	!	+	+	-	!	-

2007_Moreira_Junior	hamstrings maximum ROM	1	!	+	+	+	!	!
2008_Monteiro	bench press 10RM	1	!	+	+	!	!	!
2008_Monteiro	smith machine squat 10RM	1	!	+	+	!	!	!
2008_Monteiro	goniometric assessment of shoulder flexion	1	!	+	+	!	!	!
2008_Monteiro	goniometric assessment of shoulder extension	1	!	+	+	!	!	!
2008_Monteiro	goniometric assessment of horizontal shoulder adduction	1	!	+	+	!	!	!
2008_Monteiro	goniometric assessment of horizontal shoulder abduction	1	!	+	+	!	!	!
2008_Monteiro	goniometric assessment of elbow flexion	1	!	+	+	!	!	!
2008_Monteiro	goniometric assessment of hip flexion	1	!	+	+	!	!	!
2008_Monteiro	goniometric assessment of hip extension	1	!	+	+	!	!	!
2008_Monteiro	goniometric assessment of knee flexion	1	!	+	+	!	!	!
2008_Monteiro	goniometric assessment of trunk extension	1	!	+	+	!	!	!
2008_Monteiro	goniometric assessment of trunk flexion	1	!	+	+	!	!	!
2009_Kasser	ankle dorsiflexion ROM	1	!	+	+	+	!	!
2009_Potier	eccentric hamstrings strength 1RM	1	!	+	+	-	!	-
2009_Potier	passive knee extension test	1	!	+	+	-	!	-
2010_Santos	bench press 1rm	1	!	+	+	+	!	!
2010_Santos	goniometric assessment of shoulder flexion	1	!	+	+	+	!	!
2010_Santos	goniometric assessment of shoulder extension	1	!	+	+	+	!	!
2010_Santos	goniometric assessment of horizontal shoulder adduction	1	!	+	+	+	!	!
2010_Santos	goniometric assessment of horizontal shoulder abduction	1	!	+	+	+	!	!
2010_Santos	goniometric assessment of trunk flexion	1	!	+	+	+	!	!
2010_Santos	goniometric assessment of trunk extension	1	!	+	+	+	!	!
2011_Kim	shoulder press 1RM	1	!	+	+	!	!	!
2011_Kim	chest press 1RM	1	!	+	+	!	!	!
2011_Kim	leg press 1RM	1	!	+	+	!	!	!
2011_Kim	low row 1RM	1	!	+	+	!	!	!

2011_Kim	lat pulldown 1RM	1	!	+	+	!	!	!
2011_Kim	sit and reach test	1	!	+	+	!	!	!
2011_Simao	bench press 10rm	1	!	+	+	+	!	!
2011_Simao	leg press 10rm	1	!	+	+	+	!	!
2011_Simao	sit and reach test	1	!	+	+	+	!	!
2016_Fukuchi	MVIC of hip abductors	1	+	+	+	+	!	!
2016_Fukuchi	MVIC of hip extensors	1	+	+	+	+	!	!
2016_Fukuchi	MVIC of ankle plantar extensors	1	+	+	+	+	!	!
2016_Fukuchi	goniometric assessment of hip adduction ROM	1	+	+	+	+	!	!
2016_Fukuchi	goniometric assessment of hip external rotation ROM	1	+	+	+	+	!	!
2016_Fukuchi	goniometric assessment of hip internal rotation ROM	1	+	+	+	+	!	!
2016_Fukuchi	goniometric assessment of ankle ROM focusing on the soleus muscle	1	+	+	+	+	!	!
2016_Fukuchi	goniometric assessment of ankle ROM focusing on the gastrocnemius n	1	+	+	+	+	!	!
2017_Kim	modified sit and reach	1	!	!	+	-	!	-
2017_Kim	back scratch test	1	!	!	+	-	!	-
2017_Leite	sit and reach	1	!	+	+	+	!	!
2017_Leite	goniometric assessment of shoulder flexion	1	!	+	+	+	!	!
2017_Leite	goniometric assessment of shoulder extension	1	!	+	+	+	!	!
2017_Leite	goniometric assessment of horizontal shoulder adduction	1	!	+	+	+	!	!
2017_Leite	goniometric assessment of horizontal shoulder abduction	1	!	+	+	+	!	!
2017_Leite	goniometric assessment of elbow flexion	1	!	+	+	+	!	!
2017_Leite	goniometric assessment of hip flexion	1	!	+	+	+	!	!
2017_Leite	goniometric assessment of hip extension	1	!	+	+	+	!	!
2017_Leite	goniometric assessment of knee flexion	1	!	+	+	+	!	!
2017_Leite	goniometric assessment of trunk extension	1	!	+	+	+	!	!
2017_Leite	goniometric assessment of trunk flexion	1	!	+	+	+	!	!
2017_Smith	handgrip	1	!	+	+	+	!	!

2017_Smith	sit and reach test	1	!	+	+	-	!	-
2018_Abdel-aziem	hamstrings eccentric peak torque, 60°/s	1	+	+	+	+	!	!
2018_Abdel-aziem	hamstrings eccentric peak torque, 120°/s	1	+	+	+	+	!	!
2018_Abdel-aziem	hamstrings concentric peak torque, 60°/s	1	+	+	+	+	!	!
2018_Abdel-aziem	hamstrings concentric peak torque, 120°/s	1	+	+	+	+	!	!
2018_Abdel-aziem	9090 test, goniometric assessment of knee flexion	1	+	+	+	-	!	-
2019_Solà_Serrabou	modified sit and reach	1	!	+	+	-	!	-
2020_Baker	handgrip	1	+	!	-	+	+	-
2020_Baker	sit and reach	1	+	!	-	+	+	-
2020_Baker	back scratch	1	+	!	-	+	+	-
2006_Simons	lat pulldown 1RM	1	!	+	+	+	!	!
2006_Simons	chest press 1RM	1	!	+	+	+	!	!
2006_Simons	upper back machine 1RM	1	!	+	+	+	!	!
2006_Simons	leg extension 1RM	1	!	+	+	+	!	!
2006_Simons	leg curl 1RM	1	!	+	+	+	!	!
2006_Simons	leg press 1RM	1	!	+	+	+	!	!
2006_Simons	sit and reach	1	!	+	+	+	!	!
2006_Simons	goniometric assessment of hip flexion	1	!	+	+	+	!	!
2006_Simons	goniometric assessment of shoulder flexion	1	!	+	+	+	!	!
2006_Simons	goniometric assessment of shoulder abduction	1	!	+	+	+	!	!
2020_Piraua	handgrip Right hand	1	+	+	+	+	!	!
2020_Piraua	handgrip left hand	1	+	+	+	+	!	!
2020_Piraua	sit and reach	1	+	+	+	+	!	!
2021_Elsangedy	1RM of bench press	1	+	+	+	+	!	!
2021_Elsangedy	1RM of leg press	1	+	+	+	+	!	!
2021_Elsangedy	1RM of lat pulldown	1	+	+	+	+	!	!
2021_Elsangedy	1RM of leg extension	1	+	+	+	+	!	!

2021_Elsangedy	1RM of lateral shoulder raises	1	+	+	+	+	!	!
2021_Elsangedy	1RM of leg curl	1	+	+	+	+	!	!
2021_Elsangedy	1RM of biceps arm curl	1	+	+	+	+	!	!
2021_Elsangedy	1RM of triceps pushdown	1	+	+	+	+	!	!
2021_Elsangedy	knee extensors peak torque	1	+	+	+	+	!	!
2021_Elsangedy	knee flexors peak torque	1	+	+	+	+	!	!
2021_Elsangedy	handgrip	1	+	+	+	+	!	!
2021_Elsangedy	chair sit and reach	1	+	+	+	-	!	-
2021_Elsangedy	back scratch	1	+	+	+	-	!	-
2021_Elsangedy	passive knee extension test	1	+	+	+	+	!	!
2021_Elsangedy	single hip flexion ROM	1	+	+	+	+	!	!
2021_Elsangedy	bilateral hip flexion ROM	1	+	+	+	+	!	!
2013_Yaprak	isometric back extension strength	1	!	+	+	-	!	-
2013_Yaprak	inclinometer lumbar flexion	1	!	+	+	-	!	-
2013_Yaprak	inclinometer lumbar extension	1	!	+	+	-	!	-
2013_Yaprak	inclinometer thoracic flexion	1	!	+	+	-	!	-
2013_Yaprak	inclinometer thoracic extension	1	!	+	+	-	!	-
2017_Ribeiro-alvares	isometric peak torque of knee flexors	1	!	+	+	+	!	!
2017_Ribeiro-alvares	concentric peak torque of knee flexors	1	!	+	+	+	!	!
2017_Ribeiro-alvares	eccentric peak torque of knee flexors	1	!	+	+	+	!	!
2017_Ribeiro-alvares	chair sit and reach	1	!	+	+	-	!	-
2018_Fritz	Isometric upright row	1	-	+	+	+	!	-
2018_Fritz	Isometric squat	1	-	+	+	+	!	-
2018_Fritz	Isometric trunk extension	1	-	+	+	+	!	-
2018_Fritz	Back scratch	1	-	+	+	+	!	-
2018_Fritz	chair sit and reach	1	-	+	+	+	!	-
2021_Vatovec	passive straight leg raise rom	1	!	+	+	-	!	-

+ Low risk

! Some concerns

- High risk

D1 Randomisation process

D2 Deviations from the intended interventions

D3 Missing outcome data

D4 Measurement of the outcome

D5 Selection of the reported result

Table 2c - ROBINS-I summary

paper ID	Outcome(s)	OVERALL	D1	D2	D3	D4	D5	D6	D7
2002_Barbosa	Sit and reach	serious	serious	low	low	low	moderate	serious	ni
2002_Rogers	Handgrip	serious	serious	low	low	low	low	moderate	ni
2002_Rogers	back scratch, sit and reach	serious	serious	low	low	low	low	moderate	ni
2005_Nobrega	Handgrip	serious	serious	low	low	low	low	moderate	ni
2005_Nobrega	1RM tests	serious	serious	low	low	low	low	moderate	ni
2005_Nobrega	Flexitest	serious	serious	low	low	low	low	moderate	ni
2007_Peixoto	hamstrings max ROM	critical	critical	low	low	low	low	serious	ni
2011_Junior	Sit and reach	serious	serious	ni	low	low	low	low	ni
2011_Junior	5RM tests	serious	serious	ni	low	low	low	low	ni
2011_Morton	goniometric tests	critical	critical	low	low	low	ni	serious	ni
2011_Morton	knee flex/ext peak torque	critical	critical	low	low	low	ni	moderate	ni
2013_daCosta	fleximetry	critical	serious	low	low	low	critical	serious	ni
2013_Takeshima	back scratch, sit and reach	serious	serious	low	low	low	low	moderate	ni
2021_Versic	sit and reach, shoulder mobility	serious	serious	moderate	low	low	low	moderate	ni
2021_Versic	Handgrip	serious	serious	moderate	low	low	low	moderate	ni

D1: Bias due to Confounding; D2: Selection of participants into the study; D3: Classification of intervention; D4: Deviations from intended intervention; D5: Missing data; D6: Measurement of outcome data; D7: Selection of reported of result
