# Supplementary material for: The Influence of Resistance Training on Joint Flexibility in Healthy Adults: A Systematic Review, Meta-analysis, and Meta-regression
Source: J Strength Cond Res. 2024 Dec 31;39(3):386–97. doi: 10.1519/JSC.0000000000005000 (PMC11841725; doi:10.1519/JSC.0000000000005000)
Supplement: SUPPLEMENTARY MATERIAL [file jscr-39-0386-s004.pdf]

Table 1d - Meta-regression results table

Variable	Levels	N of ES	Estimate	95% CI	
**Year**		169			
	Intercept		27.21	-19.29	73.71
	Year		-0.01	-0.04	0.01
**Randomization**		168			
	No		0.45	0.13	0.77
	Yes		0.70	0.52	0.88
**Duration**		169			
	Intercept		0.55	0.21	0.88
	Duration		0.01	-0.02	0.03
**Sex**					
	Males	87	0.76	0.48	1.03
	Females	46	0.80	0.54	1.05
	Both	36	0.39	0.15	0.63
**Activity level**					
	Sedentary	110	0.70	0.50	0.91
	Active	42	0.45	0.08	0.83
**Baseline differences**					
	No	76	0.76	0.56	0.97
	Yes	21	0.49	-0.02	1.00
	Unknown/ unclear	72	0.48	0.23	0.73
**N. Of exercises**		167			
	Intercept		0.69	0.36	1.02
	N. of ex.		-0.01	-0.05	0.03
**Volume**		167			
	Intercept		0.64	0.39	0.89

	Volume		0.0001	-0.01	0.01
**Volume per exercise**		167			
	Intercept		0.74	0.52	0.95
	Volume per ex.		-0.02	-0.05	0.01
**Mismatch**					
	No	106	0.53	0.35	0.72
	Yes	63	0.73	0.55	0.92
**Outcome unit**					
	Cm	41	0.58	0.38	0.79
	degrees	123	0.71	0.51	0.90
**Progression**					
	No	5	0.52	-0.15	1.18
	Yes	153	0.69	0.52	0.85
	Unknown/ Unclear	11	0.24	-0.24	0.72
**Timing (per week)**					
	2	27	0.53	0.25	0.82
	3	130	0.74	0.55	0.94
	4	4	0.50	-0.29	1.29
	5	3	0.52	-0.19	1.23
	6	5	-0.08	-0.86	0.70
**Delivery**					
	Unsupervised	30	0.53	0.16	0.90
	Supervised	109	0.66	0.46	0.86
	Unknown/ unclear	30	0.64	0.25	1.04
**Intensity**					
	Low	13	0.28	-0.08	0.65

	Moderate	83	0.63	0.41	0.86
	High	33	0.75	0.50	1.00
**Age**		167			
	Intercept		0.56	0.21	0.92
	Age		0.001	-0.01	0.01
**Rest**		138			
	Intercept		0.28	0.02	0.54
	Rest		0.003	0.001	0.01
**Body part**					
	Ankle	4	0.67	0.14	1.19
	Back scratch	8	0.37	-0.01	0.74
	Elbow	12	0.53	0.19	0.87
	Hip	30	0.41	0.16	0.65
	Knee	15	0.80	0.48	1.11
	Shoulder	42	0.73	0.49	0.98
	Sit and reach	29	0.56	0.33	0.78
	Trunk	23	1.08	0.78	1.38
	Other	6	0.73	0.27	1.19

N of ES: number of unique effect sizes available for each level of each variable; Intercept: Meaningful for continuous variables, hypothetical values setting the variable value to 0.
